# An Efficient Tetraplex Surveillance Tool for Salmonid Pathogens

**DOI:** 10.3389/fmicb.2022.885585

**Published:** 2022-04-21

**Authors:** Ulla von Ammon, Tessa Averink, Karthiga Kumanan, Cara L. Brosnahan, Xavier Pochon, Kate S. Hutson, Jane E. Symonds

**Affiliations:** ^1^Aquaculture & Marine Biosecurity, Cawthron Institute, Nelson, New Zealand; ^2^College of Science and Engineering, James Cook University, Townsville, QLD, Australia; ^3^Animal Health Laboratory, Ministry for Primary Industries, Upper Hutt, New Zealand; ^4^Institute of Marine Science, University of Auckland, Warkworth, New Zealand

**Keywords:** droplet digital PCR, multiplex assay, *Oncorhynchus tshawytscha*, Chinook salmon, aquatic animal health, fish disease

## Abstract

Fish disease surveillance methods can be complicated and time consuming, which limits their value for timely intervention strategies on aquaculture farms. Novel molecular-based assays using droplet digital Polymerase Chain Reaction (ddPCR) can produce immediate results and enable high sample throughput with the ability to multiplex several targets using different fluorescent dyes. A ddPCR tetraplex assay was developed for priority salmon diseases for farmers in New Zealand including New Zealand *Rickettsia*-like organism 1 (NZ-RLO1), NZ-RLO2, *Tenacibaculum maritimum*, and *Yersinia ruckeri*. The limit of detection in singleplex and tetraplex assays was reached for most targets at 10^−9^ ng/μl with, respectively, NZ-RLO1 = 0.931 and 0.14 copies/μl, NZ-RLO2 = 0.162 and 0.21 copies/μl, *T. maritimum =* 0.345 and 0.93 copies/μl, while the limit of detection for *Y. ruckeri* was 10^−8^ with 1.0 copies/μl and 0.7 copies/μl. While specificity of primers was demonstrated in previous studies, we detected cross-reactivity of *T. maritimum* with some strains of *Tenacibaculum dicentrarchi* and *Y. ruckeri* with *Serratia liquefaciens*, respectively. The tetraplex assay was applied as part of a commercial fish disease surveillance program in New Zealand for 1 year to demonstrate the applicability of tetraplex tools for the salmonid aquaculture industry.

## Introduction

Aquaculture production has grown 5-fold over the last three decades with a total of 82.1 million tons of aquatic animals produced in 2018, making it the fastest growing food sector in the world (Boyd and McNevin, 2015; FAO, 2020). Measuring by resource requirements of production, aquaculture can achieve a significantly reduced carbon footprint and is therefore considered a sustainable food source for the growing world population (Hai et al., 2018; Boyd et al., 2020; MacLeod et al., 2020). Atlantic salmon and rainbow trout are ranked first and second for global production volume for farmed marine finfish species, respectively (FAO, 2020), making salmonids one of the most successfully farmed fishes (Iversen et al., 2020). In New Zealand, Chinook or king salmon, *Oncorhynchus tshawytscha*, is one of the highest valued aquaculture products with a total annual revenue of NZ$ 254 million and growing (Aquaculture New Zealand, 2021a,b).

Aquaculture growth and rapid domestication of aquatic organisms come with an increased risk of diverse diseases triggered through elevated stress levels due to stocking densities and potential suboptimal environmental conditions (e.g., due to sea temperature rise or anomalies; Bateman et al., 2021; Feidantsis et al., 2021). For example, more than 20 potentially pathogenic taxa (viruses, bacteria, and parasites) have been recorded from farmed *O. tshawytscha* in New Zealand among which NZ *Rickettsia*-like organisms, *Tenacibaculum maritimum*, and *Yersinia ruckeri* (serogroup O1b) are actively managed through preventative measures such as controlled area notices and vaccines (Lane et al., 2020).

Fish disease surveillance usually involves fish, water, or sediment samples sent to commercial laboratories for testing using bacteriology, virology, histology, and molecular assays (Khor et al., 2021). A variety of molecular tools have been developed for disease surveillance from fish samples. For example, fluorescence *in situ* and other hybridization techniques have been used to screen for proliferative kidney disease (PKX) in salmonids (Morris et al., 2000); DNA microarrays coupled with conventional PCR have been used for herpesvirus and pathogenic *Flavobacterium* species in fish (Lievens et al., 2011), RNA viruses were targeted with reverse transcriptase PCR in shrimp (Poulos and Lightner, 2006) and many-related technologies such as real-time PCR, loop mediated isothermal amplification (LAMP), random amplified polymorphic DNA (RAPD), restriction/amplified fragment length polymorphism (R/AFLP), and genotyping have been used for pathogen detection in aquaculture (Kim et al., 2017). Overall, efficient, sensitive, and cost-effective methods guarantee competitiveness on the national and international market (Bozzi et al., 2021; Mordecai et al., 2021) but need to overcome complicated and timely protocols leading to inefficient real-life applicability (Law et al., 2014).

Novel molecular-based assays using droplet digital Polymerase Chain Reaction (ddPCR) can produce immediate results and high sample throughput without compromising detection sensitivity (Miotke et al., 2014; Cao et al., 2020). While traditional PCR technologies are currently applied by New Zealand’s Ministry for Primary Industries (MPI) for salmon aquaculture surveillance (Brosnahan, 2020), the advantages of ddPCR are now fully acknowledged and targeted assays are being used for routine monitoring and commercial applications across medicine to biosecurity (Rowlands et al., 2019; Wood et al., 2019; Kiefer et al., 2020; Lewin et al., 2020; Netzer et al., 2021; Orioles et al., 2022). The digital droplet PCR system QX100/QX200 (Bio-Rad, California, United States) is based on partitioning each sample (e.g., extracted DNA from fish tissue) into approximately 20,000 individual droplets, with each small reaction volume containing a single target DNA fragment, which minimizes inhibition (Mazaika and Homsy, 2014; Nathan et al., 2014). Additionally, partitioning into droplets enables absolute quantification of the targeted gene fragments to be conducted through direct measurement of DNA copy numbers, removing the need for replicates and standard curve extrapolation (Hindson et al., 2011). Finally, the possibility to multiplex several target genes into a single ddPCR reaction through two optical channels and adjusting the fluorescence signal of the different targets allows for significant time and cost savings when large datasets are being processed (Hughesman et al., 2016; Lewin et al., 2020).

Endemic pathogens have been identified as immediate and emerging concerns for New Zealand’s salmon aquaculture industry, given its freedom from exotic and notifiable disease agents (Aquaculture New Zealand, 2021a,b). For example, in 2015, up to 70% of salmon summer mortalities in the Marlborough Sounds of New Zealand were associated with bacterial pathogens including New Zealand *Rickettsia*-like organism (NZ-RLO) and *T. maritimum* (see Brosnahan et al., 2016). At least two strains of NZ-RLO have been associated with clinically diseased fishes; NZ RLO1, which shares 100% homology with Tasmanian RLO, and NZ-RLO 2 which is suggested to be the more virulent of the two strains (Brosnahan et al., 2019). *Tenacibaculum maritimum* is a Gram-negative filamentous bacterium that causes ulcerative skin disease, tenacibaculosis, and has been associated with high mortality in marine fishes (Avendaño-Herrera et al., 2006; Chapela et al., 2017). Virulence is likely associated with extreme environmental conditions such as high water temperatures and/or co-infections (Diggles, 2016). Another bacterial pathogen of concern in New Zealand *O. tshawytscha* farms is an endemic strain of *Y. ruckeri* (serotype O1b), which causes enteric red-mouth disease. This bacterium originates in freshwater hatcheries but can persist in fish following transfer to marine farms (Tobback et al., 2007; Chapela et al., 2018; Lewin et al., 2020). Collectively, these four bacteria cause disease responsible for multi-billion dollar losses globally (Assefa and Abunna, 2018); therefore, cost-effective diagnostics tools are required to enable early detection and appropriate management responses to outbreaks (Brosnahan, 2020).

The aim of this study was to design and validate a novel ddPCR tetraplex assay for priority salmon diseases for use in commercial applications. The exemplar species used for the assay included New Zealand *Rickettsia*-like organism 1 (NZ-RLO1), NZ-RLO2, *T. maritimum*, and *Y. ruckeri*. Following validation, the tetraplex assay was applied as part of a commercial fish disease surveillance program in New Zealand for 1 year to demonstrate the applicability of tetraplex tools for the salmonid aquaculture industry.

## Materials and Methods

### gBlock™ Development and Bacterial Isolates

A ddPCR assay was developed for four pathogens that are known to infect farmed *O. tshawytscha* in New Zealand: New Zealand *Rickettsia*-like organism 1 (NZ-RLO1), New Zealand *Rickettsia*-like organism 2 (NZ-RLO2), *T. maritimum*, and *Y. ruckeri* serotype O1b. For ddPCR assay validation, synthetic gene fragments (gBlocks™) of each targeted gene region with specific primer and probe binding sites for the four pathogens were designed (see Supplementary Table 1) and purchased from Integrated DNA Technologies (IDT™, Singapore). Specifically, sequences from in-house cultured NZ-RLO1 and NZ-RLO2 were used as well as sequences deposited in GenBank including LC475109.1 *T. maritimum* CF3 gene for 16S ribosomal RNA, partial sequence, and NR_119063.1 *Y. ruckeri* strain ATCC 29473 16S ribosomal RNA, partial sequence (See Supplementary Table 1). Diluted gBlocks™ served further as positive controls for consistency of the assays’ performance, together with negative controls that were included in all individual runs.

Further positive testing occurred on extracted DNA from pure bacterial cultures of NZ-RLO1, NZ-RLO2, *T. maritimum,* and *Y. ruckeri*. The reference isolate of *T. maritimum* for this study was isolated in-house from the skin of New Zealand farmed *O. tshawytscha* exhibiting clinical ulcerative disease. The bacterial isolate was then tested and confirmed by PCR using the primers described by Fringuelli et al. (2012). Reference cultures for *Y. ruckeri* [W11_2108 #14b (serotype O1b)], NZ-RLO1 (IDC W15_494 10Sp), and NZ-RLO2 (IDC W16_237) were obtained from the Ministry for Primary Industries (MPI). Single colonies of *T. maritimum* and *Y. ruckeri* were used for genomic DNA extraction. The frozen culture of NZ-RLO1 and NZ-RLO2 in Epithelioma papulosum cyprini (EPC) derived from a skin tumor of carp cells was centrifuged at 6,000 rpm for 30 min to collect a pellet. The collected pellet was subsequently used for the genomic DNA extraction.

All colonies were then extracted by adding 180 μl Qiagen lysis buffer (ATL) and 20 μl of Proteinase K and incubated at 56°C for a minimum of 3 h and further processed following the AllPrep DNA/RNA mini kit instructions (Qiagen, Hilden, Germany). The quantity and quality of extracted DNA were measured using a NanoPhotometer (Implen, Munich, Germany).

### Droplet Digital PCR Tetraplex Assay Development

#### Singleplex Assays

Species-specific primers and TaqMan^®^ probes used in the present study were designed and validated in previous publications (see Table 1). Primer and probe sequences were synthesized at IDT™ (Singapore) and applied in a singleplex ddPCR assay on a QX200 AutoDG Droplet Digital PCR System (Bio-Rad, California, United States). Different genetic regions were targeted for each pathogen; specifically, the internal transcript spacer region (ITS) for NZ-RLO1, the β-subunit of the bacterial RNA polymerase (rpoB) for NZ-RLO2, and the bacterial 16S ribosomal RNA (16S rRNA) gene for *T. maritimum* and *Y. ruckeri* (Table 1).

**Table 1:** Primers and probes used in this study for specific detection of New Zealand *Rickettsia*-like organism strain 1 (NZ-RLO1), strain 2 (NZ-RLO2), *Tenacibaculum maritimum*, and *Yersinia ruckeri*.

Target organism	Target gene	Primer/Probe sequence	bp	References
NZ-RLO1	ITS	5′-CGGTGTTGAGATATAATGTTGA-3′	79	Brosnahan, 2020
		5′-TATGATCAAGTGAATAAGTGCAT-3′		
		5′-FAM-TTGTTTTATTTAAGATAAGACTTTTTGGGG-BHQ1-3′		
NZ-RLO2	rpoB	5′-TTGATTAACTCGTTGGCAA-3′	105	Gias et al., 2018
		5′-GTAATCGACTTCACCGGTAACC-3′		
		5′-FAM-CGAATGAATACGGCTTTTTAGAAAC-BHQ1-3′		
*Tenacibaculum maritimum*	16S rRNA	5′-TGCCTTCTACAGAGGGATAGCC-3′	155	Fringuelli et al., 2012
		5′-CTATCGTTGCCATGGTAAGCCG-3′		
		5′-HEX-CACTTTGGAATGGCATCG-BHQ1-3′		
*Yersinia ruckeri* O1b	16S rRNA	5′-AACCCAGATGGGATTAGCTAGTAA-3′	247	Carson and Wilson, 2009
		5′-GTTCAGTGCTATTAACACTTAACCC-3′		
		5′-HEX-AGCCACACTGGAACTGAGACACGGTCC-3′		Ghosh et al., 2016

*ITS, Internal transcript spacer region; rpoB, the β-subunit of the bacterial RNA polymerase; 16S rRNA, bacterial 16S ribosomal RNA gene. Probes were labeled with either 6-Carboxyfluorescein (FAM) or Phosphoramidite (HEX) fluorescent dye on the 5′-end and a Black Hole Quencher 1 (BHQ®-1) on the 3′-end. Bp, Base pairs of the targeted gene sequence.*

The singleplex ddPCR assays were optimized using the gBlocks™ (Supplementary Table 1). The bacterial isolates were amplified under the same reagent concentrations and thermocycling conditions for all four pathogens (slightly differing from the most optimal singleplex conditions, see Supplementary Table 2). Reaction mixtures were performed in 22 μl volumes containing 10 μl ddPCR SuperMix for Probes (No dUTP; Bio-Rad, California, United States), 450 nM of each primer and FAM or HEX labelled probes, and 1 μl of template diluted to 10^−7^ ng/μl (gBlock™). The thermocycling conditions were initiated at 94°C for 3 min followed by 35 cycles of 94°C for 30 s, 52°C for 30 s, and 72°C for 1 min, with a final extension step at 72°C for 7 min. Droplet generation was carried out according to the manufacturer’s protocol.

QuantaSoft™ Analysis Pro software (version 1.0.596) was used to assign positive and negative droplets and to convert droplet counts to copies/μl. Thresholds were manually set for each run using the amplitude between negative and positive control samples.

#### Tetraplex Assay Development

The Bio-Rad QX200 ddPCR reader contains two optical fluorescence channels, in this instance for detecting FAM and HEX labeled probes. Multiplexing of more than two targets, individually labeled with either one of the two probes with different dye labels, requires segregating the droplets according to the templates. One strategy is to mix different concentrations of FAM and HEX for the third and fourth target (Hughesman et al., 2016). For example, the assay would have 100% FAM for target 1, 100% HEX for target 2, a mix of 70% FAM and 30% HEX for target 3, and a mix of 70% HEX and 30% FAM for target 4. Using these proportions, positive droplets will align orthogonally in a 2-D amplitude display (Supplementary Figure 1). The second strategy explores a staggered layout in the 2-dimensional display that can be reached by adjusting amplitude fluorescence using different primer and probe concentrations and additionally profiting from the different length of the targeted amplicons (Dobnik et al., 2016), ranging in this study from 79 to 247 bp.

The optimized 22 μl reaction volume for the tetraplex assay therefore consisted of 5 μl ddPCR Multiplex SuperMix for probes (Bio-Rad), 450 nM of all four primer combinations (see Table 1), and varying probe concentrations: 450 nM of the NZ-RLO1 FAM labeled probe, 900 nM of the NZ-RLO2 FAM labeled probe, 220 nM of the *T. maritimum* HEX labeled probe, and 450 nM of the *Y. ruckeri* HEX labeled probe. The thermocycling conditions were then adjusted to 95°C for 10 min, 40 cycles of 95°C for 30 s, 54°C for 1 min, and a final enzyme deactivation step at 98°C for 10 min.

### Tetraplex Assay Sensitivity, Inhibition, and Specificity Testing

To define the limit of detection, the tetraplex ddPCR assay was then evaluated for sensitivity running the assay on 10-fold dilution series of each gBlock™ starting from 10 to 10^−9^ ng/μl (see Table 2). Additionally, the assay was run individually and for all targets combined on undiluted bacterial isolates that were extracted from cultures but were of insufficient DNA quality for further dilutions and inhibition experiments. Copies/μl between individual and combined targets were compared to detect sensitivity loss between the singleplex and tetraplex assays.

**Table 2:** Droplet digital PCR results on singleplex and tetraplex assay in copies/μl on a 10-fold dilution series of NZ-RLO1, NZ-RLO2, *Tenacibaculum maritimum*, and *Yersinia ruckeri* gBlocks™ starting from 10 ng/μl.

gBlock™ concentration (ng/μl)	Singleplex (copies/μl)				Tetraplex (copies/μl)			
	NZ-RLO1	NZ-RLO2	*Tenacibaculum maritimum*	*Yersinia ruckeri*	NZ-RLO1	NZ-RLO2	*Tenacibaculum maritimum*	*Yersinia ruckeri*
10	>10^6^	>10^6^	>10^6^	>10^6^	>10^6^	>10^6^	>10^6^	>10^6^
1	>10^6^	>10^6^	>10^6^	>10^6^	>10^6^	>10^6^	>10^6^	>10^6^
0.1	>10^6^	>10^6^	>10^6^	>10^6^	>10^6^	>10^6^	>10^6^	>10^6^
0.01	>10^6^	>10^6^	>10^6^	>10^6^	>10^6^	>10^6^	>10^6^	>10^6^
10^−3^	>10^6^	>10^6^	>10^6^	>10^6^	>10^6^	>10^6^	>10^6^	>10^6^
10^−4^	>10^6^	>10^6^	>10^6^	>10^6^	>10^6^	>10^6^	>10^6^	>10^6^
10^−5^	6,285	6,113	8,115	1,088	695	70.6	5,289	1,118
10^−6^	337	386	1,062	162	163	0.8	582	5.49
10^−7^	37.2	24.7	59.9	5.38	10.3	1.06	25.1	1.43
10^−8^	3.14	3.2	5.14	1.00	0.84	0	25.9	0.07
10^−9^	0.931	0.162	0.349	0	0.14	0.21	0.93	0
10^−10^	0	0	0	0.08	0	0	0	0

*>10^6^ = too high template concentration to quantify copy numbers*.

**Figure 1:**
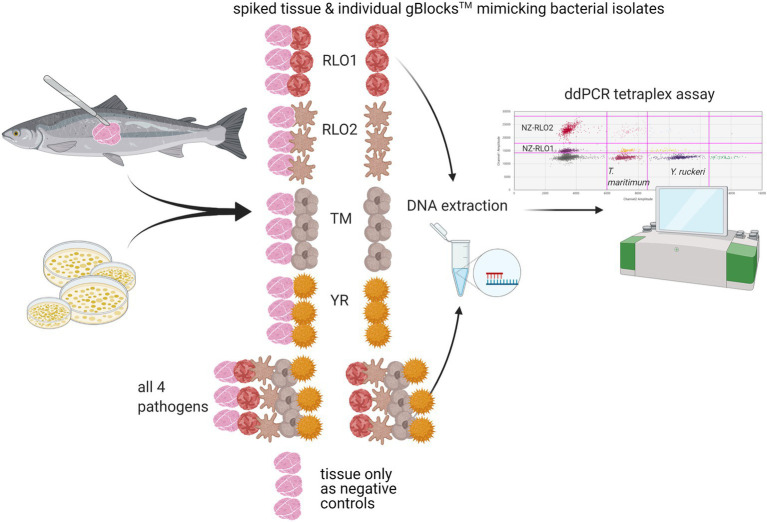
Tetraplex assay sensitivity testing on triplicate samples of DNA from *Oncorhynchus tshawytscha* tissue spiked with gBlocks™ representing either New Zealand *Rickettsia*-like organism 1 (NZ-RLO1), NZ-RLO2, *Tenacibaculum maritimum,* and *Yersinia ruckeri,* and triplicate samples of gBlocks™ with no and just *Oncorhynchus tshawytscha* DNA were included for each pathogen individually and all together. The tetraplex assay was then run for the extracted DNA of all samples and copies/μl compared between samples. Schematic created with BioRender.com.

A spiking experiment to check inhibition through fish tissue was performed on gBlocks™. New Zealand farmed *O. tshawytscha* were sourced from a freshwater salmon farm, where three of the marine pathogens, NZ-RLO1, NZ-RLO2, and *T. maritimum* have not been detected. Approximately 30 mg of fish skin with muscle was dissected under sterile conditions for DNA extraction (as described in section “gBlock™ Development and Bacterial Isolates”) and tested negative for all four pathogens using the developed tetraplex assay prior to the experiment. Triplicate samples of 30 mg clean *O. tshawytscha* tissue were spiked with gBlocks™ from NZ-RLO1, NZ-RLO2, *T. maritimum,* and *Y. ruckeri* individually and with all four in combination (see Figure 1). Negative tissue controls were included and the ddPCR tetraplex assay was performed as described under section “gBlock™ Development and Bacterial Isolates”. Droplet digital PCR copies/μl were then assessed for each of the samples.

Additionally, the tetraplex assay was run against DNA extracted from pure bacterial isolates relevant to New Zealand marine aquaculture, to ensure no cross-reactivity occurred (see Supplementary Table 3).

### Commercial Application

The ddPCR tetraplex assay was applied as part of a commercial disease surveillance program for a New Zealand salmon farming company for 12 months. Tests were conducted for approximately 30 fish samples per month (i.e., more than 360 fish in total) from up to 10 different locations including fish of freshwater and marine origin. The salmon company determined which locations were sampled each month. Tissue received for testing (pooled anterior kidney, spleen, and liver) was preserved in DNA/RNA-Shield™ isolation buffer (Zymo Research, United States). Once received by the laboratory, samples were stored at −20°C. For DNA extraction, the pooled tissue of each individual fish was subsampled to 30 mg and placed into 180 ATL buffer and 20 μl of Proteinase K and incubated at 56°C for 3 h and further processed as previously described. DNA was eluted into 100 μl and stored at −20°C until the ddPCR tetraplex assay was run, maximizing sample numbers to increase economic efficiency. The commercial testing included bacteriological plating techniques as in (Kumanan et al., 2020, in prep.) and conventional PCR techniques from MPI for cross-validation of representative samples.

## Results

### Singleplex and Tetraplex Assay Sensitivity Comparison

**Figure 2:**
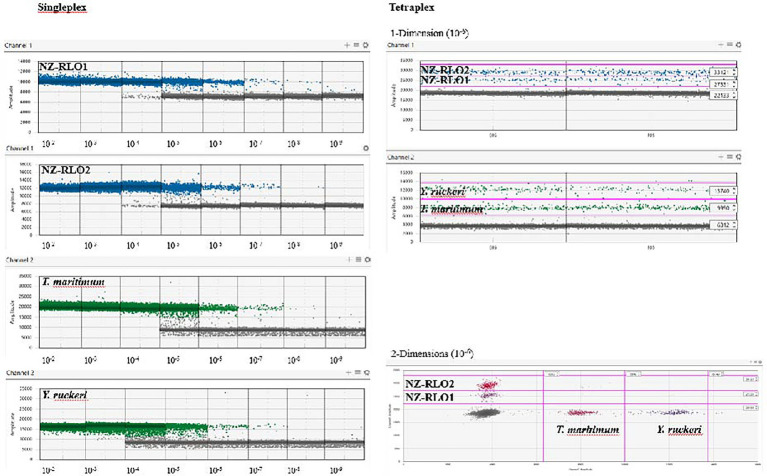
Droplet digital PCR results on singleplex (left) and tetraplex (right) assays visualized using the QuantaSoft™ Analysis Pro software (version 1.0.596) on a 10-fold gBlocks™ dilution series for NZ-RLO1, NZ-RLO2, *Tenacibaculum maritimum*, and *Yersinia ruckeri*, starting from 10 ng/μl. Tetraplex outputs are only displayed for concentration of 10^−6^ ng/μl. Blue and green dots are positive droplets on the FAM and HEX channel, respectively. Gray dots are counted as negative droplets.

Droplet digital PCR singleplexing and tetraplexing detected all four salmonid pathogens; New Zealand *Rickettsia*-like organism 1 (NZ-RLO1), New Zealand *Rickettsia*-like organism 2 (NZ-RLO2), *T. maritimum*, and *Y. ruckeri* in a reproducible and quantitative manner. The limit of detection in the singleplex and tetraplex assay was reached for most targets at 10^−9^ ng/μl with, respectively, NZ-RLO1 = 0.931 and 0.14 copies/μl, NZ-RLO2 = 0.162 and 0.21 copies/μl, *T. maritimum =* 0.345 and 0.93 copies/μl, while the limit of detection for *Y. ruckeri* was 10^−8^ with 1.0 copies/μl and 0.7 copies/μl (Table 2, Figure 2). Optimal droplet separation obtained a strong signal for each pathogen derived from the gBlocks™ dilution series at 10^−6^ ng/μl in singleplex and at 10^−7^ ng/μl in tetraplex and was used further for assay optimization and as positive controls (see Table 2).

**Figure 3:**
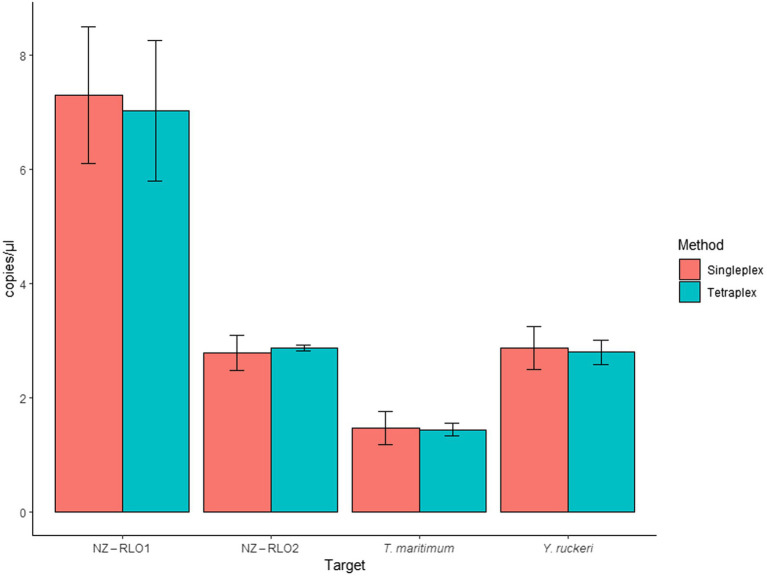
Droplet digital PCR singleplex and tetraplex assays run for bacterial isolates for NZ-RLO1, NZ-RLO2, *Tenacibaculum maritimum*, and *Yersinia ruckeri* in copies/μl. Error bars describe the standard error (SE) of the triplicate runs.

When the assay was tested as single- and tetraplex on bacterial isolates, signals resulted in 7.29 (SE = 1.19) and 7.20 (SE = 1.23) copies/μl for NZ-RLO1, 2.77 (SE = 0.3) and 2.87 (SE = 0.05) copies/μl for NZ-RLO2, 1.46 (SE = 0.29) and 1.43 (SE = 0.11) copies/μl for *T. maritimum* and 2.87 (SE = 0.37) and 2.79 (SE = 0.21) copies/μl for *Y. ruckeri* O1b, with no significant differences between the assays (Figure 3).

### Inhibition and Specificity Test

**Figure 4:**
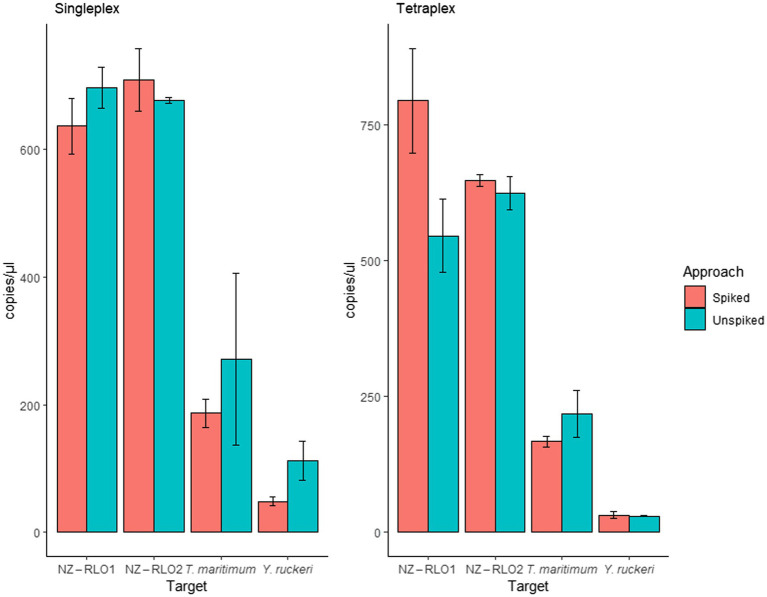
Droplet digital PCR results on singleplex and tetraplex assays run for gBlocks™ of NZ-RLO1, NZ-RLO2, *Tenacibaculum maritimum,* and *Yersinia ruckeri* individually and in combination as well as on unspiked clean (red) and spiked (blue) *Oncorhynchus tshawytscha* samples. Error bars describe the standard error (SE) of the triplicate runs.

*Oncorhynchus tshawytscha* DNA that was spiked with the gBlock™ individually (singleplex) did not reveal any inhibitory effects. Mean signals of unspiked and spiked samples resulted in 696 (SE = 32.2) and 636 (SE = 43.5) copies/μl for NZ-RLO1, 676 (SE = 4.5) and 709 (SE = 49.1) copies/μl for NZ-RLO2, 271 (SE = 135.0) and 186 (SE = 21.6) copies/μl for *T. maritimum* and 112 (SE = 30.6) and 48 (SE = 6.5) copies/μl for *Y. ruckeri*, respectively. Tetraplexing the four pathogens showed no sign of inhibition between unspiked and spiked samples. Values ranged between 545 (SE = 67.1) and 793 (SE = 96.5) copies/μl for NZ-RLO1, 623 (SE = 30.0) and 646 (SE = 11.4) copies/μl for NZ-RLO2, 217 (SE = 42.7) and 166 (SE = 10.2) copies/μl for *T. maritimum* and 30 (SE = 1.46) and 31 (SE = 6.59) copies/μl for *Y. ruckeri,* respectively (Figure 4).

Running the ddPCR tetraplex assay on extracted DNA of aquaculture relevant in-house pathogens revealed some cross-reactivity of the assay with *Serratia liquefaciens* for the *Y. ruckeri* primer and probe set. Further investigation showed that one base at the 5′-end of the *Y. ruckeri* forward primer should be A instead of G and was designed on a potentially incorrectly deployed sequence in GenBank (i.e., NR_119063.1 *Y. ruckeri* strain ATCC 29473 16S ribosomal RNA and partial sequence) and could be corrected to increase specificity. The ddPCR tetraplex assay also cross-reacted between *T. maritimum* and 3 out of 14 tested *T. dicentrarchi* strains (copy numbers = 0.13–35, Supplementary Table 3).

### Commercial Testing

All pathogens were detected from commercial samples undergoing routine disease surveillance screening and were confirmed as true positives using alternative techniques including media plating, biochemical tests, PCR, and sequencing (see Supplementary Table 3). Of the 360 fish tested, nine tested positive for NZ-RLO1, five for NZ-RLO2, 146 for *T. maritimum*, and 5 for *Y. ruckeri*.

## Discussion

The ddPCR tetraplex assay developed in this study provides a rapid, cost-effective, and reliable screening tool for four primary aquaculture pathogens with commercial relevance and application. Other studies have proven multiple pathogens to be screened in single real-time PCR assays and their implementation as a cost-efficient disease monitoring tool (Chapela et al., 2018; Peters et al., 2018).

For this molecular diagnostic tool to become an accredited test, it needs to undergo appropriate validation (Thalinger et al., 2020). The primers and probes used in this study have been previously published and checked for sensitivity and specificity (see Table 1 and references therein) and were then optimized to work in combination with a ddPCR instrument. While the approach with different probe concentrations (i.e., the assay having 100% FAM for target 1, 100% HEX for target 2, a mix of 70% FAM and 30% HEX for target 3, and a mix of 70% HEX and 30% FAM for target 4) revealed good separation between the target organisms (Supplementary Figure 1), we found an amplitude assay was easier for pipetting purposes and clearer result output especially when dealing with unclean signals (“rain”) from a degraded sample. *In vitro* testing on reference tissue was achieved by successfully amplifying reference cultures of the pathogens individually and in combination. Additionally, gBlocks™ were spiked into uncontaminated, presumably healthy, fish tissue DNA to check for any inhibitory effects. No inhibition was detected. This test was applied commercially for salmon farm surveillance for 12 months in conjunction with additional validation *via* bacterial culture techniques and standard and qPCR confirmatory analysis by MPI. Successful and true positive detections were achieved using our tetraplex assay on field samples that also aligned with other studies, e.g., *T. maritimum* detections in Bateman et al. (2021).

Diagnostic tools can never achieve 100% sensitivity and specificity (Assefa and Abunna, 2018). For example, cultivation-based methods will only detect bacteria that are able to replicate under the provided conditions and most aquatic microorganisms (>99%) are unable to be cultivated using standard methods (Netzer et al., 2021). NZ-RLO1 and NZ-RLO2 are fastidious, and good-quality DNA extracts from cultures could not be achieved for molecular purposes; thus, artificially constructed DNA oligos (gBlocks™) were used for assay optimization. By combining these screening technologies, cross-validating outcomes and interpreting them in the context of their designed application, developed tools need to be continuously optimized.

During this study, three important observations gave us novel insights for New Zealand aquaculture surveillance. First, the *T. maritimum* specific primers and probe used in this study revealed low positive signal for a non-typical *T. maritimum*-like bacterial colony on agar plates, which was then confirmed as *T. dicentrarchi* through 16S rRNA gene sequencing. Fringuelli et al. (2012) did not include this strain for specificity testing as it is simply not possible to validate primers on all existing bacterial strains (Collins et al., 2006). We tested the assay on several *T. dicentrarchi* strains and received three positive signals out of 19 tested strains. Further strain confirmation by sequencing and intraspecific variability are needed to confirm cross-reactivity or wrongly identified strains/cross-contamination with *T. maritimum*.

Our second observation of cross-reactivity of *Y. ruckeri* with closely related *Serratia* species (both Yersiniaceae) was also not included as part of the specificity testing by Carson and Wilson (2009) and Ghosh et al. (2016). We discovered through a standard GenBank BLAST that the *Y. ruckeri* forward primer might have been developed on an incorrectly deposited reference sequence (NR_119063.1) that included a single base-pair mistake at the 5′-end of the sequence, dating back to 1993 when sequencing technology was in its beginnings and prone to erroneous nucleotide outputs. This theory is supported by the fact that none of the recent deposited sequences for *Y. ruckeri* identified that particular nucleotide which is located at the 3′-end of our forward primer. DNA polymerase requires the 3′ base of a primer to form appropriate hydrogen bonds to initiate polymerization and might experience loss in sensitivity through nucleotide ambiguities (van Pelt-Verkuil et al., 2008).

Finally, a high number of positive detections of NZ-RLO 1 following inactivated (DNA) vaccination on the fish farm revealed that testing DNA with the ddPCR specific assays would not be able to differentiate between a vaccinated fish or a true infection, reported previously (Laurin et al., 2020). Investigating RNA or other viability tests such as PEMAX that focus on live pathogens could help circumvent this problem at least for inactivated vaccines (Brosnahan et al., 2020).

Optimizing the tetraplex assay further will involve designing more specific primers to avoid cross-reactions between strains and correcting other primer issues, including the design of new primers for emerging agents, such as *T. dicentrarchi* and *Serratia* strains. Novel ddPCR technologies such as the QX600 AutoDG Flex from Bio-Rad are already on the market that work on four optical channels for multiplexing up to eight samples which could be used to test eight pathogens simultaneously. Overall, there is substantial interest in adopting approaches that allow for point-of-need and in-field surveillance (Peters et al., 2018). Future technologies such as the portable Oxford Nanopore Sequencing or the NS2 Nucleic Sensing System^1^ which allows for in-field (water) multi-probe ddPCR analyses are promising for these purposes.

The baseline ddPCR tetraplex assay presented here can be repurposed and adjusted to advanced technologies, including new pathogens of interest or analytical methods, which will foster scientific research output and expand the molecular surveillance toolbox. For example, applying occupancy and co-occurrence modeling approaches on the detection signals of different pathogens enables detection probabilities even if all samples return negative (Willoughby et al., 2016; Laurin et al., 2020) and provides a cost-efficient tool for aquaculture surveillance (Farrell et al., 2021).

## Data Availability Statement

The original contributions presented in the study are included in the article/**Supplementary Material**, further inquiries can be directed to the corresponding author/s.

## Ethics Statement

All fish sampling was done with approval from the Nelson Marlborough Institute of Technology Animal Ethics Committee (AEC 2018 CAW01). Written informed consent was obtained from the owners for the participation of their animals in this study.

## Author Contributions

UA, JS, and XP designed the research. UA, TA, and KK performed the research. UA, CB, JS, and KH contributed the reagents and analytical tools. UA analyzed the data. UA, JS, KH, and XP wrote the paper. All authors contributed to the article and approved the submitted version.

## Funding

This work was funded by the New Zealand Ministry of Business, Innovation and Employment, under the program: Aquaculture Health to Maximise Productivity and Security (CAWX1707).

## Conflict of Interest

The authors declare that the research was conducted in the absence of any commercial or financial relationships that could be construed as a potential conflict of interest.

## Publisher’s Note

All claims expressed in this article are solely those of the authors and do not necessarily represent those of their affiliated organizations, or those of the publisher, the editors and the reviewers. Any product that may be evaluated in this article, or claim that may be made by its manufacturer, is not guaranteed or endorsed by the publisher.

## Footnotes

^1^ https://ns2co.com/
