# Supplementary material for: An Efficient Tetraplex Surveillance Tool for Salmonid Pathogens
Source: Front Microbiol. 2022 Apr 21;13:885585. doi: 10.3389/fmicb.2022.885585 (PMC9069008; doi:10.3389/fmicb.2022.885585)
Supplement: Supplementary file 2 [file Data_Sheet_1.DOCX]

Supplementary Material

# Supplementary Figures and Tables

**
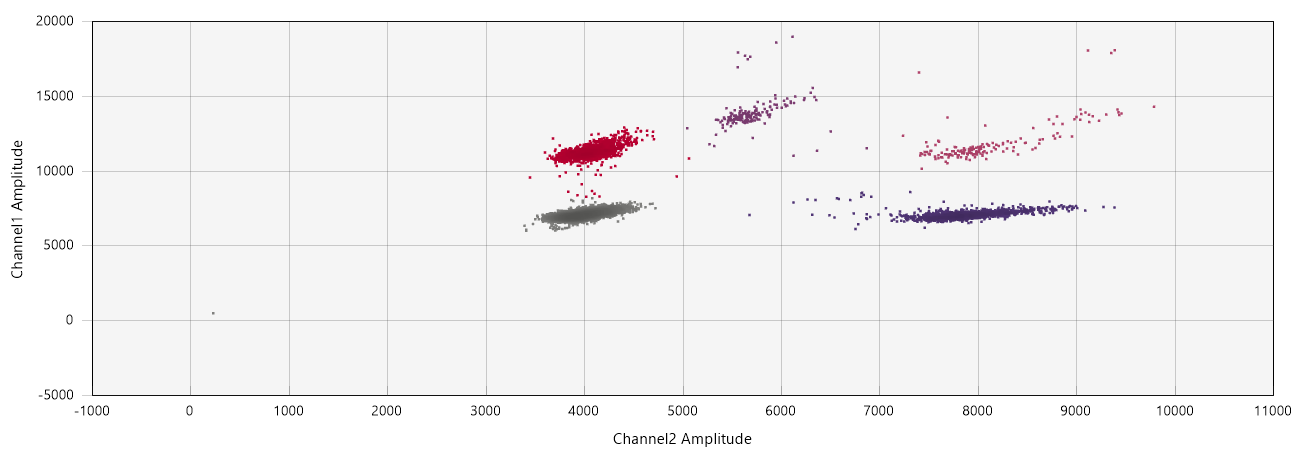
**

NZ-RLO1

NZ-RLO2

*T. maritimum*

*Y. ruckeri*

Supplementary Figure 1: Orthogonal display of ddPCR tetraplexing achieved by mixing FAM and HEX probe ratios in the two optical channels. The separation was achieved by 100% FAM for NZ-RLO11, 100% HEX for *Y. ruckeri*, a mix of 70% FAM and 30% HEX for *T. maritimum* and a mix of 70% HEX and 30% FAM for NZ-RLO2.

Supplementary Table 1: gBlocks^TM^ designed on reference sequences for all four bacterial pathogens (New Zealand *Rickettsia*-like organism 1 (NZ-RLO1) and 2 (NZ-RLO2), *Tenacibaculum maritimum* and *Yersinia ruckeri*) with primer and probe binding sites marked in yellow and pink, respectively. Random nucleotides from the original sequence were changed in the gBlock^TM^ in case of laboratory cross-contamination and are marked in green.

| **NZ-RLO1:** |
| --- |
| In-house sequence (Dr Cara Brosnahan): |
| TTGTTGATTTTATTGTTTAGTGAGAATGATATTTGTTCTTTAACAATGTGGTAAAAAGTATAAGTAAAGATTCCTTGATTAATTTAGGGTTATTTTTAGTTTTGATTGAGATGTATTTATATGTCTTGATTGATAATTGAGAATAATTTTTAGTTTATTTAATTAACGAGTCTTGGTAATTTTTGAAAACCGGTGTTGAGATATAATGTTGATTTGTTTTATTTAAGATAAGACTTTTTGGGGTTATATGATCAAGTGAATAAGTGCATA |
| RLO1- gBlocks^TM^: TTAGTTTTGATTGAGATGTATTTATATGTCTTGATTGATAATTGAGAATAATTTTTAGTTTATTTAATTAACGAGTCTTGGTAATTTTTTGTTGATTTTATTGTTTAGTGAGAATGATAGCTGTTCTTTAACAATGTGGTAAGCAGTATAAGTAAAGATTCCTTGATTAATTTAGGGTTATTTTGAAAACCGGTGTTGAGATATAATGTTGAATTGTTTTATTTAAGATAAGACTTTTTGGGGAATTATGATCAAGTGAATAAGTGCATA |
| **NZ-RLO2:** |
| In-house sequence (Dr Cara Brosnahan): |
| CAAACATCGGTTTGATTAACTCGTTGGCAACGTACGCTAGAACGAATGAATACGGCTTTTTAGAAACGCCGTATCGTCGAGTTGCTGATGGCAAGGTTACCGGTGAAGTCGATTACTTATCTGCGATTGAAGAAGCTCGGCATATTATTGCTCAGGCCAATGCGACTGTCGGTGAAGACGGTAGCTTAACCGATAGTTTATTGCACTGTCGTCAATCAGGTGAGACCTTCTTTACCACGGCGGATAAAGTCGATTATATGGACGTTGCTCCGCGCCAGATGGTCTCTGTGGCCGCGTCGATGATTCCGTTCTTAGAACACGATGATGCGAACCGGGCCTTGATGGGATCGAACATGCAACGCCAAGCAGTGCCGACCTTAATTT |
| RLO2- gBlocks^TM^: |
| CAAACATCGGTTTGATTAACTCGTTGGCAACGTACGCGCTAACGAATGAATACGGCTTTTTAGAAACGCCGTATCGTCGAGAACCTGATGGCAAGGTTACCGGTGAAGTCGATTACTTATCTGCGATTGAAGAAGCTCGGCATATTATTGCTCAGGCCAATGCGACTGTCGGTGAAGACGGTAGCTTAACCGATAGTTTATTGCACTGTCGTCAATCAGGTGAGACCTTCTTTACCACGGCGGATAAAGTCGATTATATGGACGTTGCTCCGCGCCAGATGGTCTCTGTGGCCGCGTCGATGATTCCGTTCTTAGAACACGATGATGCGAACCGGGCCTTGATGGGATCGAACATGCAACGCCAAGCAGTGCCGACCTTAATTT |
| ***Tenacibaculum maritimum*:** |
| LC475109.1 *Tenacibaculum maritimum* CF3 gene for 16S ribosomal RNA, partial sequence |
| GAATCTGCCTTCTACAGAGGGATAGCCTTTAGAAATGAAGATTAATACCTCATAACACTTTGGAATGGCATCGTTTTAAAGTTAAAGATTTATCGGTAGAAGATGACTATGCGTCCTATTAGCTAGATGGTAAGGTAACGGCTTACCATGGCAACGATAGGTAG |
| *Tenacibaculum maritimum* gBlocks^TM^: |
| GAATCTGCCTTCTACAGAGGGATAGCCTTTAGATTAGAAGATTAATACCTCATAACACTTTGGAATGGCATCGTTTTAAAGTTAAAGATTTATCGGTAGAAGATGACATAGCGTCCTATTAGCTAGATGGTAAGGTAACGGCTTACCATGGCAACGATAGGTAG |
| ***Yersinia ruckeri:*** |
| NR_119063.1 *Yersinia ruckeri* strain ATCC 29473 16S ribosomal RNA, partial sequence |
| ATGAACCCAGATGGGATTAGCTAGTAAGTGGGGTAATGGCTCACCTAGGCGACGATCCCTAGCTGGTCTGAGAGGATGACCAGCCACACTGGAACTGAGACACGGTCCAGACTCCTACGGGAGGCAGCAGTGGGGAATATTGCACAATGGGCGCAAGCCTGATGCAGCCATGCCGCGTGTGTGAAGAAGGCCTTCGGGTTGTAAAGCACTTTCAGCGAGGAGGAAGGGTTAAGTGTTAATAGCACTGAACATTGA |
| *Yersinia ruckeri* gBlocks^TM^: |
| GATGAACCCAGATGGGATTAGCTAGTAAGTCCCCTAATGGCTCACCTAGGCGACGATCCCTAGCTGGTCTGAGAGGATGACCAGCCACACTGGAACTGAGACACGGTCCAGACTCCTACCCCAGGCAGCAGTGGGGAATATTGCACAATGGGCGCAAGCCTGATGCAGCCATGCCGCGTGTGTGAAGAAGGCCTTCGGGTTGTAAAGCACTTTCAGCGAGGAGGAAGGGTTAAGTGTTAATAGCACTGAACATTGA |

Supplementary Table 2: Polymerase chain reaction (PCR) conditions after original publications (see references in Table 1) and singleplex droplet digital PCR conditions as developed in this study for all four bacterial pathogens (New Zealand *Rickettsia*-like organism 1 (NZ-RLO1) and 2 (NZ-RLO2), *Tenacibaculum maritimum* and *Yersinia ruckeri*).

| Target  (gene) |  | NZ-RLO1 (ITS) | NZ-RLO2 (rpoB) | *T. maritimum*  (16S rRNA) | *Y. ruckeri*  (16S rRNA) |
| --- | --- | --- | --- | --- | --- |
| Original PCR conditions | Primer/probe concentration (nM)  Annealing Temperature (°C)  Number of thermocycles | 220  60  45 | 220/90  60  50 | 220  52  45 | 205/45  60  40 |
| Singleplex ddPCR conditions (this study) | Primer/probe concentration (nM)  Annealing Temperature (°C)  Number of thermocycles | 450  54  45 | 450  56  40 | 450  56  40 | 450  56  35 |


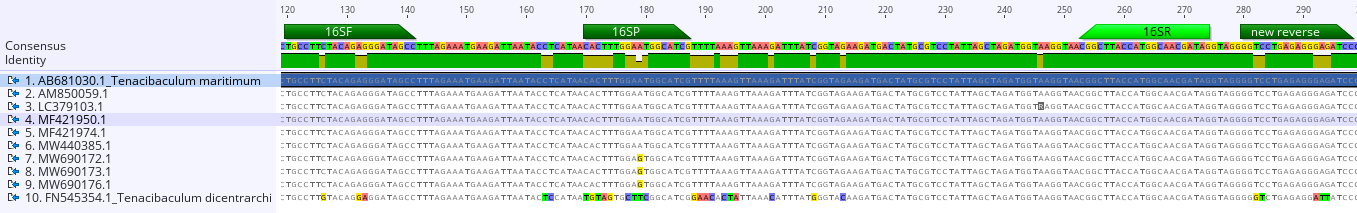


Supplementary Figure 2: Geneious alignment of 16S rRNA *Tenacibaculum maritimum* and *T. dicentrarchi* sequences and the forward and reverse primers and probe used in this study as well as a new suggested reverse primer.


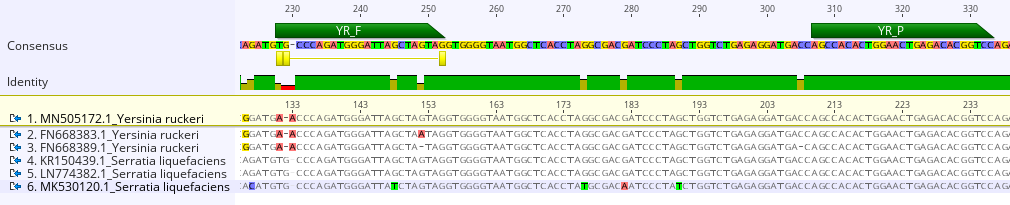

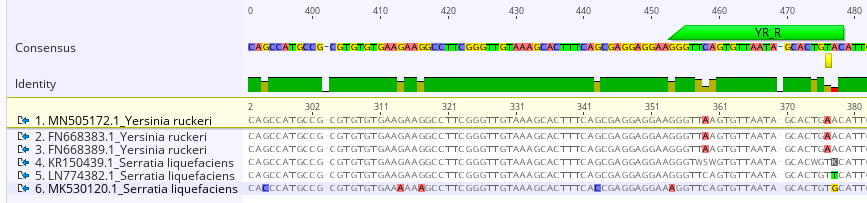


Supplementary Figure 3: Geneious alignment of the 16S rRNA *Yersinia ruckeri* and *Serratia liquefaciens* sequences and the forward and reverse primers and probe used in this study.

Supplementary Table 3: List of bacterial isolates from direct *Oncorhynchus tshawytscha or Perna canaliculus* tissue (when labeled) or in-house culture collections at the Cawthron Institute or from the Institute of Environmental Science and Research (ESR) used for specificity testing of the ddPCR tetraplex assay (in copies/µL).

| List of bacterial species | Host | NZ-RLO1 | NZ-RLO2 | *T. maritimum* | *Y. ruckeri* |
| --- | --- | --- | --- | --- | --- |
| NZ-RLO1 (NZ MPI) | *O. tshawytscha* | 0.7 | 0 | 0 | 0 |
| NZ-RLO2 (NZ MPI) | *O. tshawytscha* | 0 | 1.24 | 0 | 0 |
| *Tenacibaculum maritimum* (CCCM 001) | *O. tshawytscha* | 0 | 0 | 743 | 0 |
| *Yersinia ruckeri* (NZ MPI) | *O. tshawytscha* | 0 | 0 | 0 | >10^6^ |
| *Escherichia coli* (NZRM 916) | *NA* | 0 | 0 | 0 | 0 |
| *Yersinia enterocolitica* (NZRM 2603) | *NA* | 0 | 0 | 0 | 0 |
| *Serratia aureus* (NZRM 917) | *NA* | 0 | 0 | 0 | 0 |
| *Tenacibaculum dicentrarchi* (CCCM157) | *P. canaliculus* | 0 | 0 | 0 | 0 |
| *Tenacibaculum dicentrarchi* (CCCM 158) | *P. canaliculus* | 0 | 0 | 0 | 0 |
| *Tenacibaculum dicentrarchi* (CCCM 159) | *P. canaliculus* | 0 | 0 | 0.13 | 0 |
| *Tenacibaculum dicentrarchi* (CCCM 134) | *O. tshawytscha* | 0 | 0 | 0 | 0 |
| *Tenacibaculum dicentrarchi* (CCCM 135) | *O. tshawytscha* | 0 | 0 | 0.15 | 0 |
| *Tenacibaculum dicentrarchi* (CCCM 138) | *O. tshawytscha* | 0 | 0 | 0 | 0 |
| *Tenacibaculum dicentrarchi* (CCCM 142) | *O. tshawytscha* | 0 | 0 | 0 | 0 |
| *Tenacibaculum dicentrarchi* (CCCM 143) | *O. tshawytscha* | 0 | 0 | 0 | 0 |
| *Tenacibaculum dicentrarchi* (CCCM 146) | *O. tshawytscha* | 0 | 0 | 35 | 0 |
| *Tenacibaculum dicentrarchi* (CCCM 148) | *O. tshawytscha* | 0 | 0 | 0 | 0 |
| *Tenacibaculum dicentrarchi* (CCCM 149) | *O. tshawytscha* | 0 | 0 | 0 | 0 |
| *Tenacibaculum dicentrarchi* (CCCM 151) | *O. tshawytscha* | 0 | 0 | 0 | 0 |
| *Tenacibaculum dicentrarchi* (CCCM 030) | *O. tshawytscha* | 0 | 0 | 0 | 0 |
| *Tenacibaculum dicentrarchi* (CCCM 153) | *O. tshawytscha* | 0 | 0 | 0 | 0 |
| *Tenacibaculum soleae* (CCCM 029) | *O. tshawytscha* | 0 | 0 | 0 | 0 |
| *Tenacibaculum soleae* (CCCM 032) | *O. tshawytscha* | 0 | 0 | 0 | 0 |
| *Tenacibaculum soleae* (CCCM 023) | *O. tshawytscha* | 0 | 0 | 0 | 0 |
| *Tenacibaculum finnmarkense* (CCCM 150) | *O. tshawytscha* | 0 | 0 | 0 | 0 |
| *Tenacibaculum discolor* (CCCM 160) | *P. canaliculus* | 0 | 0 | 0 | 0 |
| *Tenacibaculum gallaicum* (CCCM 161) | *P. canaliculus* | 0 | 0 | 0 | 0 |
| *Tenacibaculum litopenaei* (CCCM 162) | *P. canaliculus* | 0 | 0 | 0 | 0 |
| *Tenacibaculum aestuarii* (CCCM 163) | *P. canaliculus* | 0 | 0 | 0 | 0 |
| *Tenacibaculum sediminilitoris* (CCCM 164) | *P. canaliculus* | 0 | 0 | 0 | 0 |
| *Tenacibaculum haliotis* (CCCM 165) | *P. canaliculus* | 0 | 0 | 0 | 0 |
| *Tenacibaculum aiptasiea* (CCCM 166) | *P. canaliculus* | 0 | 0 | 0 | 0 |
| *Flammeovirga kamogawensis* (CCCM 167) | *P. canaliculus* | 0 | 0 | 0 | 0 |
| *Flammeovirga arenaria* (CCCM 168) | *P. canaliculus* | 0 | 0 | 0 | 0 |
| *Serratia liquefaciens* (CCCM 145) | *O. tshawytscha* | 0 | 0 | 0 | >10^6^ |
| *Aeromonas sobria* (CCCM 169) | *O. tshawytscha* | 0 | 0 | 0 | 0 |
| *Aeromonas bestiarum* (CCCM 170) | *O. tshawytscha* | 0 | 0 | 0 | 0 |
| *Pseudomonas marginalis* (CCCM 171) | *O. tshawytscha* | 0 | 0 | 0 | 0 |
| *Pseudomonas putida* (CCCM 172) | *O. tshawytscha* | 0 | 0 | 0 | 0 |
| *Vagococcus salmoninarum* (CCCM 173) | *O. tshawytscha* | 0 | 0 | 0 | 0 |
| *Photobacterium piscicola* (CCCM 174) | *O. tshawytscha* | 0 | 0 | 0 | 0 |
| *Vibrio atlanticus* (CCCM 175) | *O. tshawytscha* | 0 | 0 | 0 | 0 |
| *Vibrio scophthalmi* (CCCM 176) | *O. tshawytscha* | 0 | 0 | 0 | 0 |

Supplementary Table 4: Droplet digital PCR results for the commercialized assay on NZKS samples.

Please find as separate excel file.
